# Medicare Payment to Service Volume Elasticities for Glaucoma Procedures From 2013 to 2021

**DOI:** 10.1155/joph/1780052

**Published:** 2026-07-19

**Authors:** Rita Vought, Victoria Vought, Albert S. Khouri

**Affiliations:** ^1^ Department of Ophthalmology and Visual Science, Rutgers New Jersey Medical School, Newark, New Jersey, USA, rutgers.edu

**Keywords:** glaucoma, Medicare, minimally invasive glaucoma surgery (MIGS), reimbursement policies

## Abstract

**Purpose:**

Glaucoma is a leading cause of irreversible blindness, and Medicare payments influence access to and utilization of glaucoma procedures. This study examines the relationship between Medicare payments and service volumes for glaucoma procedures.

**Methods:**

This retrospective longitudinal study used Medicare Part B National Summary Data Files from the Centers for Medicare and Medicaid Services (CMS). Data for glaucoma procedures from 2013 to 2021 with at least five years of available data were analyzed. The dataset included service volumes, average payments, total practicing ophthalmologists, and Medicare Part B beneficiary trends across the United States. The relationship between Medicare payments and service volumes was quantified using payment‐volume elasticities, calculated as the percentage change in procedural volume per 1% change in Medicare reimbursement.

**Results:**

Twenty procedures were evaluated. Minimally invasive glaucoma surgeries (MIGS) exhibited the greatest sensitivity, with a payment‐volume elasticity of 4.67% (*p* < 0.001): for every 1% increase in payment, MIGS volume increased by 4.67%. Traditional Glaucoma Surgeries (TGS) had a payment‐volume elasticity of 1.96% (*p* = 0.002), with a 1% decrease in payment resulting in a 1.96% decrease in TGS volume. Specific procedures like goniotomy (CPT: 65820) and aqueous drainage device (CPT: 66183) also showed significant elasticities of 4.27% and 4.13%, respectively.

**Conclusions:**

A significant relationship between Medicare payment adjustments and the utilization of glaucoma procedures was observed, with potential implications for access and care delivery. MIGS demonstrated the highest sensitivity to payment changes, suggesting that reimbursement policies could influence the adoption and availability of these surgeries beyond clinical decision‐making.

## 1. Introduction

With an aging population in the United States (US), the prevalence of glaucoma continues to rise [1], increasing the demand for effective management strategies. Several surgical options may be considered in the management of glaucoma. The landscape of glaucoma surgeries has undergone significant changes in recent years [2], influenced by shifts in Medicare reimbursement rates and advancements in surgical techniques. Traditional glaucoma surgeries (TGS), such as trabeculectomies and aqueous tube shunt procedures, are the conventional gold‐standard glaucoma surgeries [3]. However, the emergence of new technologies, classified under minimally invasive glaucoma surgery (MIGS), has emerged as a promising approach, offering reduced risk and faster recovery times compared to traditional options.

Significant national policy debate surrounds the Medicare reimbursement of glaucoma procedures. With overall decreasing reimbursement for many ophthalmic procedures [4], understanding trends in the utilization and reimbursement of glaucoma surgery is critical for assessing their economic impact and informing policy decisions. This study aimed to quantify Medicare service volume and payment trends as payment‐volume elasticities for common glaucoma procedures between 2013 and 2021, providing insights into the relationship between Medicare reimbursements and procedural volume.

## 2. Methods

**FIGURE 1:**
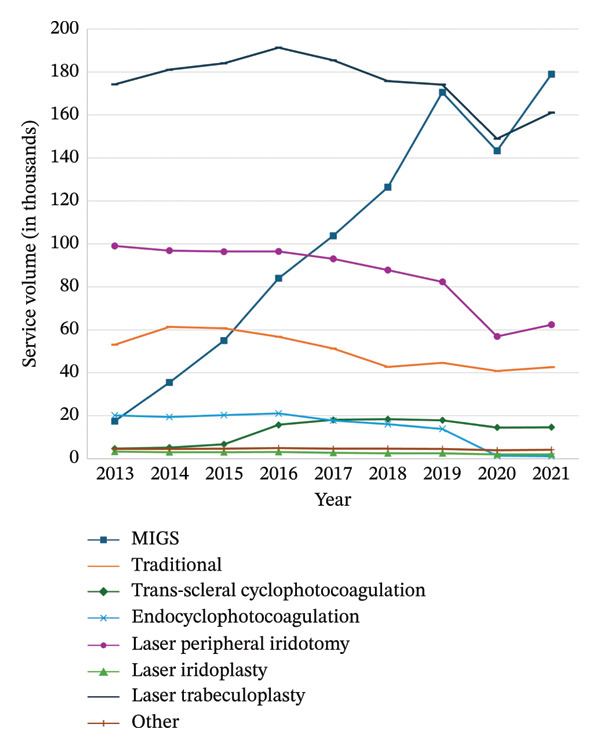
Total service volume from 2013 to 2021 by procedure type: MIGS, traditional glaucoma surgeries, trans‐scleral cyclophotocoagulation, endocyclophotocoagulation, laser peripheral iridotomy, laser iridoplasty, laser trabeculoplasty, and other.

Current Procedural Terminology (CPT) codes of common glaucoma procedures were identified [5]. CPT codes were included if they had at least 5 years of data from 2013 to 2021. Twenty CPT codes met our inclusion criteria (Table 1). CPT codes were classified as MIGS (CPT: 65820, 66174, 66175, 0191T, 0449T); TGS (CPT: 65850, 66170, 66172, 66179, 66180, 66183); trans‐scleral cyclophotocoagulation [TSCPC] (CPT: 66710); endocyclophotocoagulation [ECP] (CPT: 66711), laser peripheral iridotomy (CPT: 66761); laser iridoplasty (CPT: 66762); laser trabeculoplasty (CPT: 65855); and Other (CPT: 66184, 66185, 66625, 66630) (Figure 1) [6].

**TABLE 1:** Service volume and average payments (in 2021 USD) for common glaucoma procedures.

Procedure		2013	2014	2015	2016	2017	2018	2019	2020	2021
Goniotomy (CPT: 65820)	SV	233	159	249	4986	11,904	23,242	37,647	32,782	38,159
	AP	$717.83	$655.63	$606.46	$1050.41	$969.96	$930.55	$867.62	$968.39	$971.69

Trabeculotomy (CPT: 65850)	SV	2279	2404	2544	2623	2031	1196	869	590	412
	AP	$726.17	$678.24	$689.27	$678.54	$667.81	$674.85	$661.72	$647.84	$600.07

Laser trabeculoplasty (CPT: 65855)	SV	174,233	181,079	184,077	191,344	185,476	175,748	174,155	149,027	161,047
	AP	$293.66	$287.95	$277.83	$222.18	$185.37	$185.23	$186.60	$182.15	$170.95

Trabeculectomy (CPT: 66170)	SV	20,968	20,006	20,152	18,518	16,786	13,508	12,070	9691	9588
	AP	$944.36	$942.74	$928.97	$793.33	$849.42	$836.06	$831.07	$824.21	$771.01

Trabeculectomy, scar (CPT: 66172)	SV	8816	8182	8345	7387	6391	5192	4877	3676	3757
	AP	$1232.68	$1215.27	$1180.20	$990.80	$954.14	$936.43	$925.67	$919.09	$859.95

Canaloplasty (CPT: 66174)	SV	754	576	1578	2125	4155	8229	18,677	19,297	30,540
	AP	$1191.62	$1160.07	$1183.25	$690.25	$1111.22	$1114.72	$1109.73	$1095.65	$974.02

Canaloplasty, stent (CPT: 66175)	SV	2212	1603	1067	1033	775	514	349	409	779
	AP	$1245.60	$1235.84	$1225.09	$1236.00	$1156.69	$1144.72	$1078.50	$1109.75	$979.12

Tube shunt (CPT: 66179)	SV	N/A	N/A	683	1784	1782	1754	1429	1190	1266
	AP	N/A	N/A	$1210.07	$1243.27	$1167.50	$1167.08	$1161.72	$1158.27	$1097.42

Tube shunt, graft (CPT: 66180)	SV	20,997	21,219	20,640	19,434	18,539	17,170	17,242	15,464	16,071
	AP	$1211.59	$1190.32	$1166.86	$1176.29	$1139.71	$1119.20	$1297.00	$1324.62	$1254.13

Aqueous drainage device, external approach (CPT: 66183)	SV	N/A	9645	8404	7083	5778	3973	8100	10,215	11,531
	AP	N/A	$1192.21	$1185.86	$1188.86	$1139.00	$1130.77	$1328.95	$1407.50	$1354.11

Revise tube shunt (CPT: 66184)	SV	N/A	N/A	649	808	911	1153	1054	890	917
	AP	N/A	N/A	$639.74	$646.53	$625.92	$613.98	$592.59	$616.67	$583.13

Revise tube shunt, graft (CPT: 66185)	SV	3120	3196	2898	2981	2766	2688	2629	2356	2429
	AP	$837.96	$807.84	$723.02	$719.47	$701.60	$696.74	$694.58	$690.30	$648.32

Transscleral cyclophotocoagulation (CPT: 66710)	SV	4745	5182	6756	15,742	18,049	18,390	17,812	14,500	14,660
	AP	$417.74	$427.18	$483.11	$481.61	$465.74	$456.15	$450.42	$459.38	$428.57

Endocyclophotocoagulation (CPT: 66711)	SV	20,174	19,368	20,230	21,023	17,722	16,112	13,909	1319	1158
	AP	$392.17	$393.53	$472.89	$489.19	$479.15	$486.86	$482.35	$403.71	$395.57

Laser peripheral iridotomy (CPT: 66761)	SV	99,105	96,905	96,418	96,495	93,059	87,791	82,374	56,885	62,376
	AP	$252.92	$247.35	$240.90	$237.47	$229.79	$231.24	$232.54	$229.58	$217.90

Iridoplasty (CPT: 66762)	SV	3373	3011	3086	3223	2793	2520	2526	2058	2019
	AP	$459.36	$453.36	$444.82	$440.82	$429.58	$437.79	$443.34	$435.03	$421.21

Aqueous drainage device, internal approach (CPT: 0191 T)	SV	14,301	33,093	52,106	75,794	84,454	84,691	103,591	84,000	103,601
	AP	$1142.74	$1122.50	$1141.59	$1186.24	$1411.69	$1266.47	$1196.38	$1212.59	$1183.55

ADD, subconj (CPT: 0449 T)	SV	N/A	N/A	N/A	N/A	2539	9704	10,314	6900	5931
	AP	N/A	N/A	N/A	N/A	$1304.23	$1291.37	$1374.37	$1443.31	$1422.28

Iridectomy peripheral (CPT: 66625)	SV	1354	1366	1109	1084	877	764	859	676	773
	AP	$303.64	$307.63	$283.72	$332.64	$335.11	$330.26	$308.35	$323.68	$280.23

Iridectomy sector (CPT: 66630)	SV	55	56	86	68	83	78	79	68	91
	AP	$429.54	$422.56	$378.11	$425.33	$457.17	$386.78	$386.78	$336.41	$315.25

*Note:* AP = average payment (in 2021 USD).

Abbreviation: SV = service volume.

Medicare service volume and total payments for these procedures were sourced from the Centers for Medicare Services (CMS) Part B National Summary Data Files [7]. Average payments were calculated by dividing the total payments for a CPT code by the service volume. All payments were adjusted for inflation using the Bureau of Labor Statistics Consumer Price Index calculator [8] to USD in the year 2021. Annual Medicare Part B enrollment was determined using CMS program statistics [9]. Total practicing ophthalmologists that submitted Medicare Part B claims by year were identified using the CMS Provider Summary files [10]. Income per capita was not included in this analysis due to lack of data availability for the study period [11].

To determine the service volume response to changes in Medicare payments, a fixed‐effects regression model was utilized. Payment‐volume elasticities have been previously utilized to analyze medical procedures and are calculated as the percent change in volume per 1% change in Medicare payment [12]. This model was adapted from previous literature and accounted for yearly variation in Medicare ophthalmologists and total Medicare B beneficiaries nationally [12]. Elasticities were calculated for MIGS and TGS categories and for each procedure separately.

Statistical analyses were performed using IBM SPSS (Chicago, IL) with a statistical significance at *p* = 0.05. Institutional Review Board approval was not obtained because this research did not involve human subjects.

## 3. Results

Chronologic trends in billing varied by the type of glaucoma procedure (Figure 1). Goniotomy (CPT: 65820), canaloplasty (CPT: 66174), aqueous drainage device (ADD), subconj (CPT: 0449T), ADD (CPT: 0191T), and tube shunt (CPT: 66179) had the greatest average year‐to‐year increase in procedure volume: 278.3%, 70.9%, 60.3%, 34.1%, and 21.8%, respectively. Total volume decreased the most for Trabeculotomy (CPT: 65850), ECP (CPT: 66711), Trabeculectomy, scar (CPT: 66172), Trabeculectomy (CPT: 66170), and Canaloplasty, stent (CPT: 66175): −17.4%, −17.1%, −9.7%, −9.0%, and −5.9% per year, respectively. The overall average annual percent change in total service volume was 3.21%, while the average annual percent change in procedure payment was −0.86%.

Total MIGS procedure volume (CPT: 65820, 66174, 66175, 0191T, 0449T) increased 37.4%/year, and payments increased by 1.44%/year. Conversely, TGS volume (CPT: 65850, 66170, 66172, 66179, 66180, 66183) decreased by 3.19%/year and payments decreased as well (1.30%/year).

The fixed‐effects regression model for calculation of Medicare payment‐volume elasticity was significant for MIGS (*p* < 0.001) and TGS (*p* < 0.001), with payment‐volume elasticities of 4.67 (95% CI: 2.109 to 7.226; *p* < 0.001) and 1.96 (95% CI: 0.734 to 3.189; *p* = 0.002), respectively. For every 1% increase in payment for MIGS, volume increased by 4.67%; for every 1% decrease in payment, TGS volume decreased by 1.96% (Table 2). For MIGS, the number of ophthalmologists to volume elasticity and number of beneficiaries to volume elasticity were 6.55 (95% CI: −43.47 to 56.57; *p* = 0.791) and 12.45 (95% CI: 7.18 to 17.71; *p* < 0.01), respectively. For TGS, these elasticities were 7.09 (95% CI: −5.630 to 19.806; *p* = 0.267) and −2.62 (95% CI: −4.161 to −1.080; *p* = 0.001), respectively. Significant payment‐volume elasticities were observed for goniotomy (CPT 65820) at 4.27% (95% CI: 1.03 to 7.51; *p* = 0.019) and ADD, external approach (66183) at 4.13% (95% CI: 1.727 to 6.532; *p* = 0.009).

**TABLE 2:** Payment‐volume elasticities for glaucoma procedures in the United States.

Procedure	Category	SV change (%)	AP change (%)	Elasticity (95% CI)	Elasticity *p* value
Goniotomy (CPT: 65820)	M	278.34	6.31	4.271 (1.032–7.510)	0.019
Trabeculotomy (CPT: 65850)	T	−17.36	−2.31	−3.777 (−12.661 to 5.107)	0.324
Laser trabeculoplasty (CPT: 65855)	LTP	−0.76	−6.24	−0.120 (−0.835 to 0.595)	0.684
Trabeculectomy (CPT: 66170)	T	−9.03	−2.33	−0.745 (−2.453 to 0.963)	0.313
Trabeculectomy, scar (CPT: 66172)	T	−9.68	−4.27	−0.233 (−2.258 to 1.792)	0.779
Canaloplasty (CPT: 66174)	M	70.89	0.77	0.611 (−1.192–2.424)	0.424
Canaloplasty, stent (CPT: 66175)	M	−5.91	−2.86	−1.627 (−8.818 to 5.564)	0.586
Tube shunt (CPT: 66179)	T	21.77	−1.57	1.596 (−12.870–27.061)	0.855
Tube shunt, graft (CPT: 66180)	T	−3.18	0.61	−0.105 (−0.838 to 0.628)	−0.728
Aqueous drainage device, external approach (CPT: 66183)	T	9.23	2.06	4.130 (1.727–6.532)	0.009
Revise tube shunt (CPT: 66184)	O	7.12	−1.48	−0.736 (−0.8683–7.210)	0.787
Revise tube shunt, graft (CPT: 66185)	O	−2.94	−3.10	−0.064 (−1.154 to 1.027)	0.887
Transscleral cyclophotocoagulation (CPT: 66710)	CPC	21.06	0.46	0.608 (−4.846–6.063)	0.786
Endocyclophotocoagulation (CPT: 66711)	ECP	−17.10	0.53	3.031 (−2.645–8.707)	0.228
Laser peripheral iridotomy (CPT: 66761)	LPI	−4.92	−1.83	−3.158 (−6.557 to 0.241)	0.063
Iridoplasty (CPT: 66762)	LI	−5.89	−1.06	−1.106 (−4.699 to 2.488)	0.465
Aqueous drainage device, internal approach (CPT: 0191 T)	M	34.10	0.75	−0.108 (−4.193 to 3.978)	0.949
Aqueous drainage device, subconj (CPT: 0449 T)	M	60.33	2.25	−0.264 (−109.304 to 108.777)	0.993
Iridectomy peripheral (CPT: 66625)	O	−5.84	−0.63	−0.780 (−2.404 to 0.845)	0.272
Iridectomy sector (CPT: 66630)	O	8.96	−3.36	−0.469 (−3.327 to 2.389)	0.691

*Note:* AP = average payment (in 2021 USD), LTP = laser trabeculoplasty, ECP = endocyclophotocoagulation, CPC = cyclophotocoagulation.

Abbreviations: LI = laser iridoplasty, M = MIGS, O = other, SV = service volume, T = traditional.

## 4. Discussion

We observed an overall increase in glaucoma surgery volume, likely as a result of increasing need for such procedures in the US despite falls in inflation‐adjusted procedure reimbursement [4]. This change is a result of a multitude of factors, of which Medicare reimbursement may be influential. Medicare reimbursement rates for glaucoma procedures have been declining over the last 2 decades. Francone et al. reported a 20.5% reduction in the average adjusted reimbursement for glaucoma procedures between 2000 and 2020, with significant decreases in procedures such as laser trabeculoplasty, laser iridotomy, and cyclophotocoagulation [13]. We also observed an overall decrease in mean payment; however, these changes were not distributed evenly between procedure types. Previous analysis of claims before and after major coding and reimbursement changes, such as that of January 2022, has also found significant changes in procedural volume based on these changes, with increases in MIGS and falls in TGS [14]. Changes in CPT coding may also impact the patterns that are observed [15, 16]. Our data align with established trends.

We also note the differential elasticities for MIGS (4.67%) and TGS (1.96%) over the analyzed 8‐year period. MIGS volume has greater sensitivity to payment changes, possibly due to greater novelty and variety of surgeries. As older procedures are more established in clinical evidence, newer procedures may be more impacted by features aside from direct patient demand and provider preference. For instance, they may incur higher procedure costs to offset new training and incentivize their use. The MIGS group also saw a positive elasticity, with both increasing volume and reimbursement. MIGS elasticity in our model was mainly driven by goniotomy, which not only demonstrated high elasticity but also high absolute volume compared to other procedures. Goniotomy has recently surged in popularity for treating glaucoma in adults due to advances in MIGS, with a demonstrated safety profile and consistent results [17, 18]. Although MIGS have been growing in popularity among glaucoma providers, reimbursement changes may introduce a confounding bias. An individualized approach for each patient must be achieved—while TGS may be more suitable for those with rapidly advancing glaucoma or those with severe disease, MIGS can offer a lower risk of postoperative complications and shortened recovery times [19]. While reimbursement should not bias surgical options offered to patients, it may still be contributing to the significant shifts in surgical practices observed. Further study would be required to elucidate the impacts of these reimbursement changes and policy decisions on clinical outcomes.

Other factors outside of payments likely impact both volume and pay. For instance, the year 2020 also marked the beginning of the COVID‐19 pandemic, which likely also contributed to overall decreases in nonemergent procedures during that time period, including glaucoma surgeries. Some of the differential changes by code may also be explained by other changes in practice patterns and coding regulations. For instance, approval of the Xen gel stent in 2016 likely spurred changes in practice patterns, as its approval led to increased clinical research and evidence supporting its use [20]. This may have contributed to increased physician usage of the ab externo gel stent approach (66183) and decreasing use of the ab interno approach (0449T). In addition, CPT code 66711 (ECP) was bundled with cataract surgery codes beginning in 2020, which likely contributed to the decline in 66711 utilization around the same time [16]. CPT codes that bundled cataract surgery with ECP were excluded from this analysis due to insufficient Medicare data. This may have contributed to an observed decrease in volume for ECP. In addition, within the MIGS and TGS groups, different procedures had differential elasticities and directionality; for instance, although TGS saw decreases in payment and volume, ADD, the external approach (CPT: 66183) had increases in both with positive elasticity. Changes such as these demonstrate the complexities of characterizing relationships between payment changes and service volumes both by procedure and subgroup. In Gong et al., of the six procedures analyzed, four payment‐volume elasticities (CPT: 65855, 66170, 66172, 66180) were not statistically significant, which aligns with the present findings. Differences may reflect not only changes in these trends but also the need for a nuanced approach by policy‐makers to better grasp the consequences of Medicare payment changes [12]. A limitation to the present study is that income per capita was not included as a co‐variate in the model. Prior analyses incorporated income data derived from the Area Resources File to account for regional socioeconomic variation. However, the present study period (2013–2021) was selected to capture all Medicare payment data available at the time of data collection, for which the complete corresponding income data was not available. Since income per capita could not be consistently incorporated into the model, some of the differences observed may be attributed to socioeconomic differences influencing procedure utilization.

## 5. Conclusion

This study elucidates the impact of Medicare reimbursement changes on glaucoma procedure volume, highlighting growth in overall glaucoma procedure volume in the setting of likely increased need. MIGS exhibited higher elasticity to payment adjustments than TGS, which reflects the dynamic nature of surgical practices and technology adoption. The relationship between payments and procedural volume raises important questions about how financial incentives shape healthcare delivery patterns. Our findings underscore the broader implications of reimbursement policies on access to care, provider decision‐making, and patient access to surgical options. Future analyses may characterize payment‐volume relationships using other models to further assess these differences [14]. Analysis of whether changes in reimbursement influence the proportion of specific procedures performed rather than overall procedure volumes may also demonstrate useful insights. Additional studies may also explore other factors influencing procedure volume, such as provider preferences, evolving clinical guidelines, and patient demographics. As changes to Medicare reimbursement may also impact service volume and practice patterns in later years, greater examination of specific factors impacting these changes is warranted.

## Funding

This study did not receive any funding.

## Conflicts of Interest

The authors declare no conflicts of interest.

## Data Availability

The data that support the findings of this study are available on request from the corresponding author (Rita Vought, rv456@njms.rutgers.edu).
